# [Retracted] Tumor necrosis factor-related apoptosis-inducing ligand inhibits proliferation and induces apoptosis of prostate and bladder cancer cells

**DOI:** 10.3892/ol.2025.15354

**Published:** 2025-10-22

**Authors:** Lin Hao, Yan Zhao, Zhi-Gang Li, Hou-Guang He, Qing Liang, Zhi-Guo Zhang, Zhen-Duo Shi, Pei-Ying Zhang, Cong-Hui Han

Oncol Lett 13: 3638–3640, 2017; DOI: 10.3892/ol.2017.5922

Subsequently to the publication of the above article, an interested reader drew to the attention of the Editorial Office that the flow cytometric plots for the ‘5 ng/ml^−1^ TRAIL’ experiment in Fig. 1 looked remarkably similar to a panel in Fig. 2 (showing the results of the ‘20 ng/ml^−1^ TRAIL’ experiment), even though the gated percentages were reported to be different.

In view of the apparent anomaly in the presentation of Figs. 1 and 2 in this paper, the Editor of *Oncology Letters* has decided that this article should be retracted from the publication on account of a lack of confidence in the presented data. The authors were asked for an explanation to account for these concerns, but the Editorial Office did not receive a reply. The Editor apologizes to the readership for any inconvenience caused.

